# First evidence of yawn contagion in a wild monkey species

**DOI:** 10.1038/s41598-021-96423-3

**Published:** 2021-09-09

**Authors:** Alessandro Gallo, Anna Zanoli, Marta Caselli, Elisabetta Palagi, Ivan Norscia

**Affiliations:** 1 Department of Life Sciences and Systems Biology, University of Torino, Via Accademia Albertina 13, 10123 Torino, Italy; 2 Natural History Museum, University of Pisa, Via Roma 79, 56011 Calci, Pisa Italy; 3 Unit of Ethology, Department of Biology, University of Pisa, Via Alessandro Volta 6, 56126 Pisa, Italy

**Keywords:** Biological anthropology, Animal behaviour

## Abstract

Yawn contagion occurs when individuals yawn in response to the yawn of others (triggers). This is the first account of yawn contagion in wild geladas (*Theropithecus gelada*), a monkey species that shows yawn contagion in captivity and is organized in core units (one-male/bachelor groups) forming multilevel associations. In a population of geladas from the Kundi plateau (Ethiopia) we found that the yawning response was highest when geladas could perceive a triggering yawn, which confirms that yawn contagion is present in the wild. Yawn duration, mouth-opening degree and presence/absence of vocalisation (possibly modulating yawn detectability) did not affect the likelihood of contagion. Males and females, known to be both implicated in movement initiation within groups, were similarly powerful as yawn triggers. Instead, group membership and responder sex had a significant role in shaping the phenomenon. Yawn contagion was highest between individuals belonging to different core units and males were most likely to respond to others’ yawns. Because males have a non-negligible role in inter-group coordination, our results suggest that yawn contagion may have a communicative function that goes beyond the basic unit level.

## Introduction

Contagious yawning differs from spontaneous yawning in that one individual (responder) yawns in response to a yawn emitted by another individual (trigger)^1^. In this respect, the yawn of an individual acts as a triggering stimulus (*sensu Tinbergen*^2^). While spontaneous yawning is probably an ancient phenomenon widespread in vertebrates^3,4^, intraspecific contagious yawning is an evolutionary more recent phenomenon that has been found mostly in highly social species. Such species include mammals (domestic pig, *Sus scrofa*^5^; sheep, *Ovis aries*^6^, lions, *Panthera leo*^7^, elephant seals, *Mirounga leonina*^8^; Tonkean macaques, *Macaca tonkeana*^9^; bonobos, *Pan paniscus*^10^; chimpanzees, *Pan troglodytes*^11,12^; humans, *Homo sapiens*^13^; but see: *Pongo pygmaeus*^14^) and one bird species (budgerigars, *Melopsittacus undulates*^15^). Moreover, there are some social species in which yawn contagion is virtually absent (e.g. lemurs, *Lemur catta* and *Varecia variegata*^16^; lowland gorillas, *Gorilla gorilla gorilla*^17^). In different species, yawn contagion can be triggered both by vocalized and non-vocalized yawns^18,19^, regardless of the uni- or multi-modal nature of the stimulus^13,18,20^. Yawn contagion can be influenced by environmental/social contexts and individual factors, and it can follow a diurnal rhythm with a peak in the morning^21,22^. Moreover, the phenomenon has been found to be highest between familiar or in-group subjects (wolves^23^; bonobos^24^; chimpanzees^12^; humans^18,25^). Finally, depending on the social role played by each sex in social groups, the yawning response can increase when triggers are males (dominant in chimpanzees^11^) or females (dominant in bonobos^26^). In humans, women may respond more to others’ yawns^27,28^, although this does not apply to all cohorts^18,29^. Overall, yawn contagion seems to promote inter-individual physiological resonance^30,31^, synchronisation within social groups^7^ and coordinated daily activities^7,32^.

Geladas (*Theropithecus gelada*) are a diurnal monkey species, endemic to Ethiopia, that lives in core units (One-Male-Unit, OMU; All-Male-Unit, AMU) forming discrete multilevel associations (teams and bands^33^). The core units are characterized by high levels of grooming, low levels of aggression, and no coercive control by males over females, with females mediating group coordination with the alpha male^34^. While grooming is observed only within groups and never between groups^33^, high levels of tolerance characterize the groups forming the band, which show low aggressive levels when they occupy the same foraging areas^33^.

Geladas have been the first monkey species in which yawn contagion was demonstrated^35^. In captive geladas yawn contagion was elicited by all types of yawns (covered teeth, uncovered teeth and uncovered gums) emitted with or without vocalizations. Moreover, it was highest between subjects sharing strong bonds (measured via grooming exchange rates)^35^. So far, no study has described the phenomenon of yawn contagion in geladas, and more generally in primates, under wild conditions.

Here, we investigated yawn contagion in a population of wild geladas to verify whether the phenomenon was present in the natural setting. Moreover, we investigated the possible influence of the factors that are only assessable under wild conditions to understand if the phenomenon can act on a multilayer scale reflecting the multilevel social organization of the species. Based on the previous framework, we formulated the following predictions.

*Prediction 1—Presence of yawn contagion*. As previously reported in captivity^35^, we expected to find that individuals would be more likely to yawn when they perceived the yawn of a conspecific compared to when they did not (presence of yawn contagion).

*Prediction 2—Perceptual factors.* In line with the findings obtained in captivity^35^, we expected that also in the wild yawn contagion would be induced at comparable levels by different types of triggering yawns and regardless of the presence of vocalization.

*Prediction 3*—*Contextual and individual factors.* As it occurs in humans (characterized by a diurnal lifestyle) that show a peak of yawn contagion in the morning^22^, we expected that in geladas yawn contagion could be highest in the first part of the day (Prediction 3a). Because in primates, including captive geladas, yawn contagion has been found to be most frequent between familiar subjects^12,18,23,35^, in geladas we expected to find a higher rate of contagion within rather than between groups (Prediction 3b). Finally, the different role of each sex in managing social groups can lead to sex differences in yawn contagion^25–27^. Because in geladas both males and females play an important role in managing the social groups^34^, we expected to find no difference in yawn contagion rates between sexes (Prediction 3c).

## Materials and methods

### Species and data collection

This study was conducted on a wild gelada population from the Kundi plateau (Wof-Washa area, Ethiopia, N9°40.402′ E39°45.060′)^36^. The data collection was carried out on 16 core units (14 OMUs and 2 AMUs) via video recordings (January-May 2019 and December 2019-February 2020). For this study, 22 adult males and 47 adult females were individually recognized via long-lasting external features (i.e. sex, size, permanent signs^34^). Additional adult females (N = 24) that could not be identified with certainty (i.e., on the field or during the video analysis) were dummy coded with different labels in videos from different days. Due to the absence of yawning responses in infants and juveniles^35^, we focussed our analyses only on the adults.

The different groups were recognized by group-size, sex- and age-ratio, presence of subjects with particular signs (i.e. scars, permanent deformations) and alpha male features (i.e. shape of the red chest area^37^). For all the individuals we could determine group-membership, sex, and age class.

Data were collected on a daily basis (5 days/week) from 09:00 to 15:00. We collected about 44 h of videos (Panasonic HC-V180 Full HD optic-zoom 50x, 2csec accuracy) on the highland edges where individuals alternated social and foraging activities (distance between geladas and the observers 20-25 m). Since yawning is generally inhibited during feeding (geladas^38^; lemurs, *Lemur catta* and *Propithecus verreauxi*^39^), we collected yawning data on the groups while frequenting the highland edge to maximize the likelihood to record yawning events. We never recorded positive social interactions (i.e. grooming) between adults belonging to different groups.

### Video analyses and operational definitions

To detect the presence and timing of yawning we analysed videos via VLC (version 3.0.7.1) with extension Jump-to-Time. Yawns were present in around 24 h of videos. Over the day, 1–3 groups could temporally aggregate far away from other groups. Such splitting activity together with environmental physical barriers prevented geladas from perceiving yawns of individuals present in other group aggregations. The video framing (ranging from 5 to 15 m approximately) could cover such limited number of groups and allowed the extrapolation of yawns from videos following the all-occurrences method^40^.

Those yawns that occurred in the 3-min window around copulatory, aggressive, and human disturbance events were discarded from the analyses. For the other yawns we recorded: (i) the identity of the trigger (the individual emitting the yawn); (ii) daily time-slot; (iii) duration (seconds) and type of the triggering yawn (CT = Covered Teeth; UT = Uncovered Teeth; UG = Uncovered Gums^35^); (iv) identity of the potential responder (all individuals that were present on video); (v) sex and group membership of the trigger and the potential responder; (vi) distance between trigger and responder; (vii) presence/absence of yawn vocalization; (viii) whether the potential responder could detect the yawn; (ix) whether the potential responder yawned within 3 min from the trigger’s yawn^18,24^. A yawn emitted by the trigger was considered as not detectable when it was not vocalised and the potential responder looked away from the trigger (face of the potential responder turned 180° away from the trigger or when an obstacle prevented the potential responder from seeing the triggering yawn^25^). On a sample of 128 vocalized yawn (29 individuals) that we recorded from the study population, we determined that yawn vocalization had an intensity of 53 dB ± 10 SD (recorded at a distance of 28 m ± 16 SD). The doubtful cases were excluded from the analyses. The remaining of 90 yawns came from 31 independent individuals (18 adult males and 13 adult females) and three dummy coded adult females.

Previous studies indicate that yawning can be triggered within 5 min after perceiving a triggering yawn^1,24,35,41^. However, from the fourth minute there is a highest probability of autocorrelation (meaning that the presence of a yawn performed by a subject at t_0_ increases the probability to have another yawn by the same subject at t_(0+X)_ where X is the increasing unit of time^42^). Moreover, captive geladas showed a peak of yawn contagion in the second minute after perceiving a triggering stimulus^35^. For this reason, we selected a 3-min time window to check for the yawning response. To further reduce the autocorrelation issues, in case of a chain of yawns emitted by the trigger (more yawns emitted in the 3-min time window) we recorded as a response only the first yawn emitted after the detection of the last triggering yawn. To control for possible false positives (labelling as contagious yawns what may be—in fact—spontaneous yawns) and false negatives (labelling as spontaneous yawns what may be—in fact—contagious yawns) we also checked the yawning responses within the first and the second minute. (Supplementary Table S1). The videos were analysed by AG and 10% of the videos were also analysed by another independent coder (MC) to verify the inter-observer reliability (Cohen’s k ≥ 0.87).

### Statistics

To test for the possible presence of yawn contagion and the possible influence of different factors we ran a Generalized Linear Mixed Model (GLMM; N = 180 events perceived/not-perceived by each potential responder). The presence/absence of a yawning response in the 3 min following a trigger’s yawn was included as dependent variable (binomial, presence/absence). The factorial fixed factors included in the model were: yawn perception (yes/no); yawn type (CT; UT; UG); group-membership (same/different); distance (proximity: trigger and responder could touch one another by extending a limb; non-proximity); time-slot (09:01–12:00; 12:01–15:00); sex of the trigger and the responder (male/female); vocalisation (present/absent), trigger yawn’s duration (seconds; numeric factor). The group-identity of the dyad was included as a random factor. As a control on yawning response distribution and yawn contagion presence, two other GLMMs (with the same exact dependent variable, fixed, and random factors) were run considering a 1-min and 2-min time window (Supplementary Table S1).

We ran the GLMM in R (version 3.5.3; https://www.R-project.org) by using the function *glmer* of the R-package *lme4*^43^. Via likelihood ratio test (ANOVA with argument *Chisq*^44^, we verified if the full-model significantly differed from the null-model (only including random factors). Subsequently, with the R-function *drop1*^45^, we calculated the p-values for the individual predictors based on likelihood ratio tests between the full- and the null-model. The effect size of the significant predictors was calculated via the function *effectsize*^46^.

### Ethics approval

This research was purely observational, and no animal manipulation was required during the study. Hence, no ethical approval was necessary according to the current regulation.

## Results

**Figure 1:**
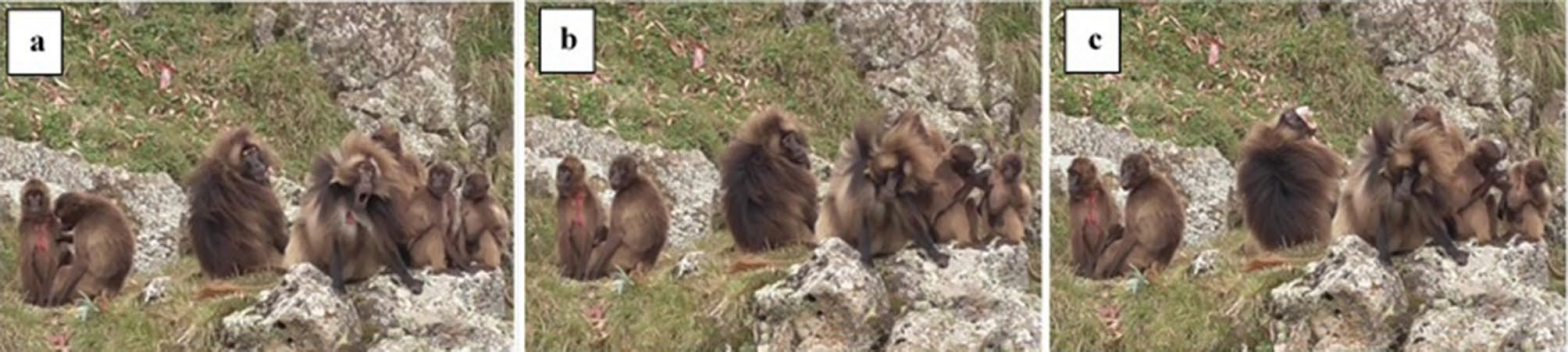
Yawn contagion in wild geladas. Yawn contagion between two alpha males belonging to two different OMUs (OMU-Sem and OMU-Cer), on the cliffs of Kundi (Ethiopia). The trigger emitted a Covered Teeth yawn (CT) (frame a; time 06:52) followed by an Uncovered Gums yawn (UG) response (frame c; time 06:56). Latency between frame a and frame c = 00:04. (Frames from video by Alessandro Gallo and Anna Zanoli, Camera: Panasonic HC-V180 Full HD optic-zoom 50x, 2csec accuracy).

**Figure 2:**
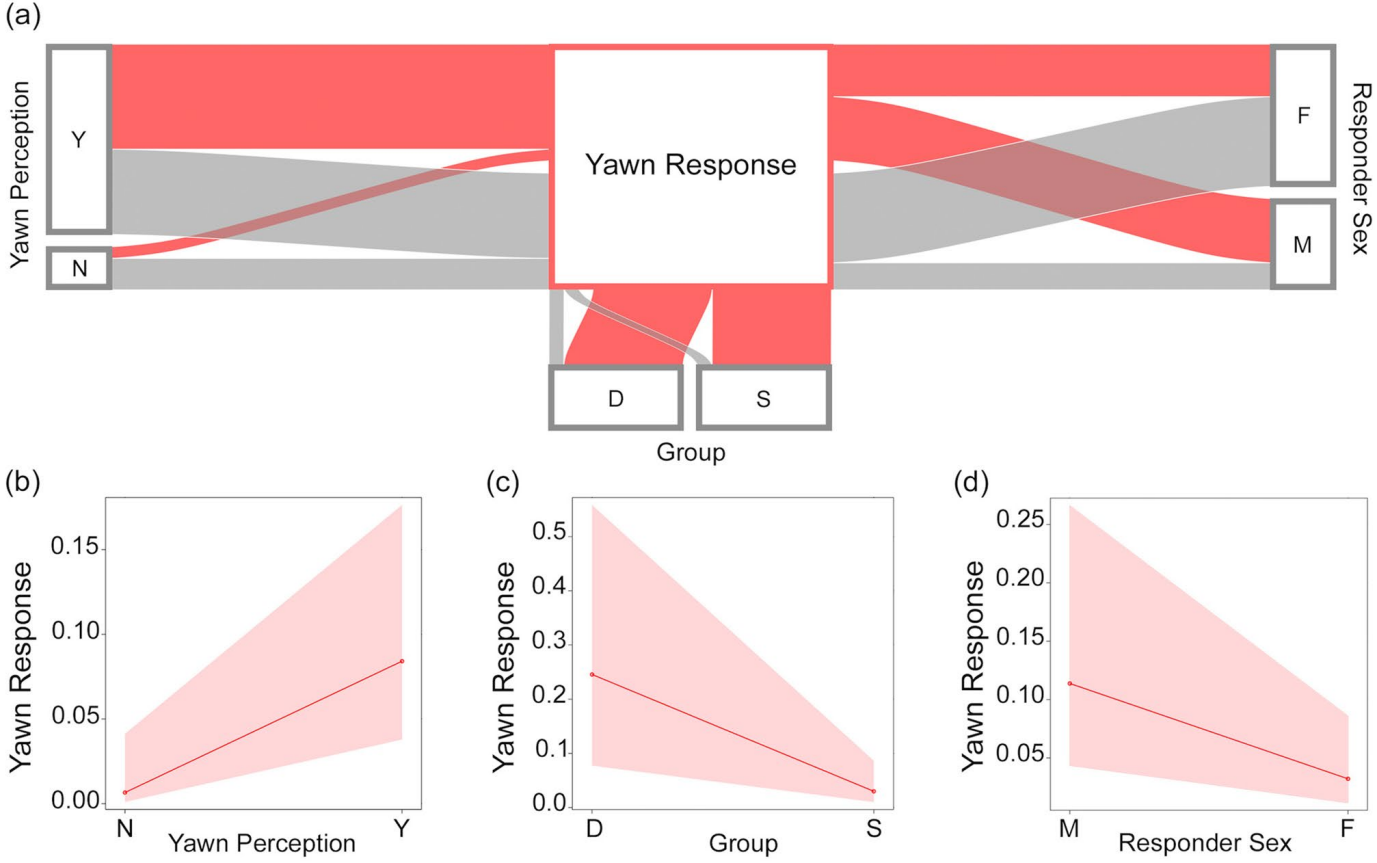
GLMM results. (**a**) Alluvial plot showing the frequency of presence (Red streams) and absence of (Grey streams) Yawn Response for each level of the factors “Perception” (Y = perception, N = no perception), “Group” (D = different groups, S = same group) and “Responder Sex” (F = female, M = male). (**b**) Effect of variables “Perception” (Y = perception, N = no perception), (**c**) “Group-membership” (D = different groups, S = same group) and (**d**) “Sex responder” (F = female, M = male) on the Yawn Response. Band represents the confidence interval. (Graphs created via R freeware, version 3.5.3; https://www.R-project.org).

**Figure 3:**
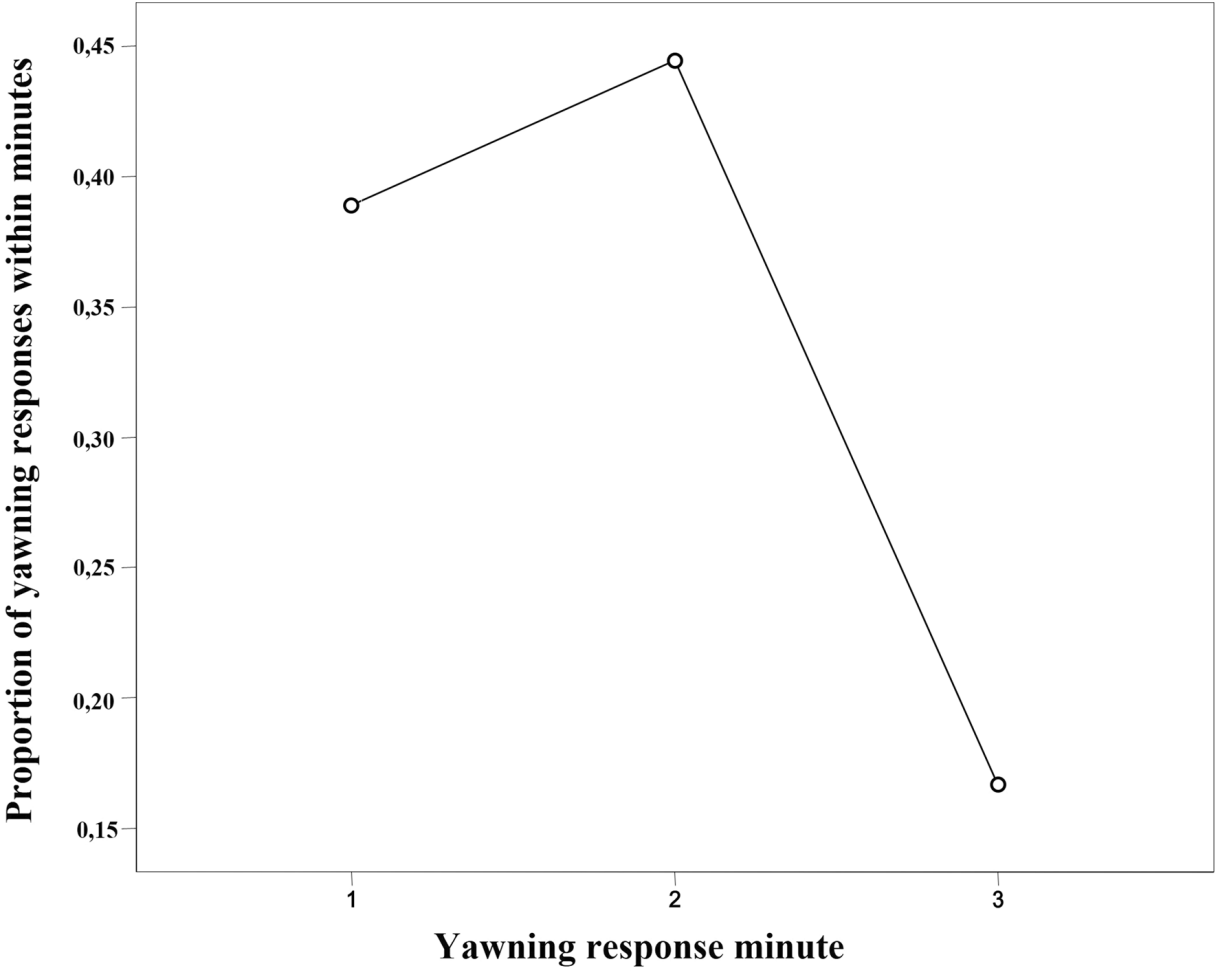
Yawn contagion distribution—proportion of yawning responses (Y axis) across the three minutes (X axis) following the triggering yawn (number of yawning responses in the 1st, 2nd, and 3rd minute over the total number yawning responses) (Graph created via SPSS, version 26).

Via GLMM, we found a significant difference between the full- and the null-model (χ^2^ = 34.0285, df = 10, p < 0.001). Stimulus perception, group membership, and sex had a significant main effect on the yawning response (Table 1; Fig. 2a). In particular, perceiving a yawn in the previous 3-min time block significantly increased the probability of a yawning response in the observer (Table 1; Figs. 1, 2b; Supplementary Video S1). Thus, yawn contagion was present in wild geladas in a 3-min time window. Considering other time windows, via two other GLMMs we found that yawn contagion could not be detected within the first minute only, whereas the phenomenon was present when considering the first two minutes from the triggering stimulus (for full results see Supplementary Table S1). As a matter of fact, 80% of the responses occurred within the second minute after the triggering stimulus. Figure 3 shows the distribution of yawn contagion across the 3 mins following the triggering stimulus.

**Table 1:** Result of the GLMM. Dependent variable = yawning performed by a subject within three minutes from others’ yawns (binomial: present = 1; absent = 0).

Fixed Effects	Estimate	SE	*z *value	*p *value	Effect size 95% CI
Intercept	− 1.387	1.836	− 0.756	^a^	–
Perception (yes)^b,c^	2.628	0.907	2.897	**0.004**	[0.85/4.41]
Group membership (same)^b,c^	− 2.354	0.968	− 2.431	**0.015**	[− 4.25/− 0.46]
Distance (non-proximity)^b,c^	− 0.740	0.823	− 0.898	0.369	–
Time slot (12:01–15:00)^b,c^	1.104	0.818	1.350	0.177	–
Sex trigger (female)^b,c^	0.357	0.858	0.416	0.678	–
Sex responder (female)^b,c^	− 1.353	0.595	− 2.272	**0.023**	[− 2.52/− 0.19]
Yawn duration	0.283	0.784	0.361	0.718	–
Yawn type (uncovered gums)^b,c^	0.014	0.720	0.019	0.985	–
Yawn type (uncovered teeth)^b,c^	− 1.781	1.202	− 1.482	0.138	
Yawn vocalization (presence)^b,c^	− 1.220	0.922	− 1.323	0.186	

Random factors = group identity of the dyad. Significance threshold: p<=0.05 Full- versus null-model: χ^2^ = 34.0285, df = 10, p < 0.001.

^a^Not shown as not having a meaningful interpretation.

^b^Estimate ± SE refers to the difference of the response between the reported level of this categorical predictor and the reference category of the same predictor.

^c^These predictors were dummy coded, with the “Perception (NO)”, “Group membership (Different)”, “Distance (Proximity)”, “Time slot (09:01–12:00)”, “Sex Trigger (Male)”, Sex Responder (Male)”, “Yawn vocalization (Absent)”, “Yawn type (Covered teeth)”, being the reference categories. Significant p-values are shown in bold.

No factor related to stimulus salience or detection (yawn type, duration, distance, presence/absence of yawn vocalisation) had a significant main effect on the yawning response (Table 1; see Supplementary Table S1 for the 2-min analysis). Similarly, the time-slot had no significant main effect (Table 1; Supplementary Table S1). Instead, the yawning response was significantly more likely between subjects belonging to a different than to the same group (Table 1; Supplementary Table S1; Fig. 2c) and in males compared to females (Table 1; Supplementary Table S1; Fig. 2d). Hence, sex and group membership, but not the salience of the stimulus, influenced yawn contagion in the wild geladas under study.

## Discussion

The present study provides the first evidence of yawn contagion in a wild primate, the gelada (in line with Prediction 1). In the considered time window, the study subjects were more likely to yawn when they could perceive a yawn from conspecifics compared to when they could not (Fig. 2b; Table 1). Most of the yawning responses occurred in the second minute, thus confirming captive data^35^. The physical distance between trigger and responder, yawn duration, and trigger yawn type (accompanied or not by vocalization) had no effect on the occurrence of the phenomenon (Table 1). Hence, the level of yawn detectability (possibly enhanced in case of long, vocalized, and wide-open yawns) appeared not to be strictly linked to the yawning response probability (in agreement with Prediction 2). This is in line with the previous report showing that all yawn types can be contagious in geladas^35^. Our data are also in agreement with results on humans that are infected by others’ yawns by having access to both total or partial view of the trigger’s face^18,20,41^. Palagi et al.^35^ found a matching of the yawn type especially in the contagion between females. We could not check for this aspect due to data limitation, but it is certainly an issue that would be worth exploring in future investigations.

As in captivity^35^, also in the wild both vocalised and non-vocalised yawns could elicit contagion at a comparable level (Table 1). This result is also consistent with findings on humans, where contagion is triggered by both the visual and acoustic components of the stimulus^19,25,47^. The acoustic component of the yawn might function when geladas are located far from the trigger and cannot visually perceive yawning. Further investigation including larger observational ranges and datasets per individual is necessary to clarify the role of multimodality on this phenomenon.

Contrary to our prediction (3b), in the study population yawn contagion was highest between individuals belonging to different groups (Fig. 2c, Table 1). Although the adult members of different groups do not engage in affiliative behaviours, they show high tolerance levels, spatial cohesion, and collective moving^33^. Considering the complexity of the gelada multilevel social system^48^, yawn contagion could be a phenomenon acting on a multilayer scale. Our limited dataset is silent on whether yawn contagion is affected by the affiliative relationships (e.g., grooming) shared by group members, thus not allowing the verification of this possibility, already tested in captivity^35^.

Contrary to the expectation (Prediction 3a), in the Kundi population the time of the day had no effect on contagion (Table 1). Because geladas arrived on the plateau relatively late in the morning (9–10 am), it was not possible to sample their behaviour soon after they started their activities. Expanding data collection to different contexts, including sleeping sites, would be crucial to determine how yawn contagion is distributed within groups according to the social affiliation shared by subjects over the 24-h cycle. However, reaching out our geladas to sample their behaviour at such sites on the cliffs may be challenging.

Finally, we found that yawn contagion: (i) was triggered to a similar extent by males and females, and (ii) males most frequently responded to others’ yawns (Fig. 2d, Table 1, Prediction 3c not supported). In the wild controlling for all the potential arousal stimuli may not be completely possible. However, our conservative exclusion of possible perturbing events (aggression, human disturbance, and copulations) and the fact that males did not preferentially respond with uncovered teeth yawns (often associated with social tension and agonistic contexts in males^38^) makes it unlikely that the response by males could be stress driven.

The trend of yawn contagion between sexes and groups suggests that this phenomenon may have a communicative function, related to activity coordination. Males and females, which were similarly powerful as yawn triggers (Table 1), can be both implicated in movement initiation within groups^34^. However, males frequently initiate the group movement, followed by the dominant female and the rest of the groups^34^. Hence, the fact that males automatically responded more frequently to others’ yawns and that yawn contagion was highest between groups, suggests that in the wild yawn contagion might also favour inter-group synchronisation during daily activities. The *Social Communicative Hypothesis* predicts that in different contexts—through yawning—individuals can involuntarily convey information about their internal state and possibly about their subsequent behaviour^32,38,39,49^. The communicative function of yawning can have an adaptive role at inter-group level (i.e., defence from predators, vigilance^50^) because it promotes the synchronization of groups’ activity through individuals that do not necessarily share strong bonds^31,51–54^. Consistently, in wild lions Casetta et al.^7^ found that yawn contagion increased coordinated movements. Hence, also in wild geladas yawn contagion may be a phenomenon that promotes the coordination within larger ecological units, such as teams and, possibly, bands^33^.

The low effect size of the significant variables affecting the yawning response (Table 1) cautions against the generalisation of the results and points toward the need of further data collection on other sites and populations. Despite this limitation, our findings open new scenarios on the role of yawn contagion in multilevel social systems. We suggest that this phenomenon operates at a multilayer scale reflecting the complex nested organization of geladas. At the basic level (core units), yawn contagion might improve synchronisation between group members sharing good relationship quality, as suggested for captive geladas^35^. In the wild, yawn contagion may also function at an upper level (team) by promoting the coordination of daily movements and activities between groups. Further studies may verify whether yawn contagion indeed elicits subsequent movements jointly started by individuals involved in the contagion not only between but also within groups (e.g.^9^).

On a broader perspective, our study underlines the importance of combining wild and captive approaches to thoroughly describe behavioural phenomena that are sensitive to different social and environmental conditions.

## Supplementary Information

Supplementary Information 1.

Supplementary Information 2.

Supplementary Video 1.

## Data Availability

The raw data supporting the conclusions of this article are provided as supporting material to the article.

## References

[1] Provine RR (2005). Yawning. Am. Sci..

[2] Tinbergen N (1952). " Derived" activities; their causation, biological significance, origin, and emancipation during evolution. Q. Rev. Biol..

[3] Baenninger R (1987). Some comparative aspects of yawning in *Betta splendens*, *Homo sapiens*, *Panthera leo*, and *Papio sphinx*. J. Comp. Psychol..

[4] Massen JJ (2021). Brain size and neuron numbers drive differences in yawn duration across mammals and birds. Commun. Biol..

[5] Norscia I, Coco E, Robino C, Chierto E, Cordoni G (2021). Yawn contagion in domestic pigs (*Sus scrofa*). Sci. Rep..

[6] Yonezawa T, Sato K, Uchida M, Matsuki N, Yamazaki A (2017). Presence of contagious yawning in sheep. Anim. Sci. J..

[7] Casetta G, Nolfo AP, Palagi E (2021). Yawn contagion promotes motor synchrony in wild lions, Panthera leo. Anim. Behav..

[8] Wojczulanis-Jakubas K, Plenzler J, Jakubas D (2019). Indications of contagious behaviours in the southern elephant seal: An observational study. Behaviour.

[9] Palagi E, Norscia I (2019). Etologia dell'emozione animale: studio e interpretazione. Sist. Intell..

[10] Amici F, Aureli F, Call J (2014). Response facilitation in the four great apes: is there a role for empathy?. Primates.

[11] Massen JJ, Vermunt DA, Sterck EH (2012). Male yawning is more contagious than female yawning among chimpanzees (*Pan troglodytes*). PLoS ONE.

[12] Campbell MW, De Waal FB (2011). Ingroup-outgroup bias in contagious yawning by chimpanzees supports link to empathy. PLoS ONE.

[13] Provine RR (1989). Faces as releasers of contagious yawning: An approach to face perception using normal human subjects. Bull. Psychonomic. Soc..

[14] van Berlo E, Díaz-Loyo AP, Juárez-Mora OE, Kret ME, Massen JJ (2020). Experimental evidence for yawn contagion in orangutans (*Pongo pygmaeus*). Sci. Rep..

[15] Gallup AC, Swartwood L, Militello J, Sackett S (2015). Experimental evidence of contagious yawning in budgerigars (*Melopsittacus undulatus*). Anim. Cogn..

[16] Reddy RB, Krupenye C, MacLean EL, Hare B (2016). No evidence for contagious yawning in lemurs. Anim. Cogn..

[17] Palagi E, Norscia I, Cordoni G (2019). Lowland gorillas (*Gorilla gorilla gorilla*) failed to respond to others’ yawn: Experimental and naturalistic evidence. J. Comp. Psychol..

[18] Norscia I, Palagi E (2011). Yawn contagion and empathy in *Homo sapiens*. PLoS ONE.

[19] Arnott SR, Singhal A, Goodale MA (2009). An investigation of auditory contagious yawning. Cogn. Affect. Behav. Neurosci..

[20] Provine RR, Heyes C, Galef B (1996). Contagious yawning and laughter: Significance for sensory feature detection, motor pattern generation, imitation, and the evolution of social behaviour. Social Learning and Animals: The Roots of Culture.

[21] Campbell MW, Cox CR (2019). Observational data reveal evidence and parameters of contagious yawning in the behavioral repertoire of captive-reared chimpanzees (*Pan troglodytes*). Sci. Rep..

[22] Giganti F, Zilli I (2011). The daily time course of contagious and spontaneous yawning among humans. J. Ethol..

[23] Romero T, Ito M, Saito A, Hasegawa T (2014). Social modulation of contagious yawning in wolves. PLoS ONE.

[24] Palagi E, Norscia I, Demuru E (2014). Yawn contagion in humans and bonobos: emotional affinity matters more than species. PeerJ.

[25] Norscia I, Zanoli A, Gamba M, Palagi E (2020). Auditory contagious yawning is highest between friends and family members: Support to the emotional bias hypothesis. Front. Psychol..

[26] Demuru E, Palagi E (2012). In bonobos yawn contagion is higher among kin and friends. PLoS ONE.

[27] Norscia I, Demuru E, Palagi E (2016). She more than he: Gender bias supports the empathic nature of yawn contagion in *Homo sapiens*. R. Soc. Open Sci..

[28] Chan MH, Tseng CH (2017). Yawning detection sensitivity and yawning contagion. Perception.

[29] Bartholomew AJ, Cirulli ET (2014). Individual variation in contagious yawning susceptibility is highly stable and largely unexplained by empathy or other known factors. PLoS ONE.

[30] De Waal FB, Preston SD (2017). Mammalian empathy: Behavioural manifestations and neural basis. Nat. Rev. Neurosci..

[31] Prochazkova E, Kret ME (2017). Connecting minds and sharing emotions through mimicry: A neurocognitive model of emotional contagion. Neurosci. Biobehav. Rev..

[32] Guggisberg AG, Mathis J, Schnider A, Hess CW (2010). Why do we yawn?. Neurosci. Biobehav. Rev..

[33] Snyder-Mackler N, Beehner JC, Bergman TJ (2012). Defining higher levels in the multilevel societies of geladas (*Theropithecus gelada*). Int. J. Primatol..

[34] Dunbar RIM (1983). Structure of gelada baboon reproductive units: IV. Integration at grouop level. Z. Tierpsychol..

[35] Palagi E, Leone A, Mancini G, Ferrari PF (2009). Contagious yawning in gelada baboons as a possible expression of empathy. PNAS.

[36] Caselli M (2021). Wild geladas (*Theropithecus gelada*) in crops—more than in pasture areas—reduce aggression and affiliation. Primates.

[37] Gallo A, Caselli M, Norscia I, Palagi E (2021). Let’s unite in play! Play modality and group membership in wild geladas. Behav. Process..

[38] Leone A, Ferrari PF, Palagi E (2014). Different yawns, different functions? Testing social hypotheses on spontaneous yawning in *Theropithecus gelada*. Sci. Rep..

[39] Zannella A, Norscia I, Stanyon R, Palagi E (2015). Testing yawning hypotheses in wild populations of two strepsirrhine species: *Propithecus verreauxi* and *Lemur catta*. Am. J. Primatol.

[40] Altmann J (1974). Observational study of behavior: Sampling methods. Behaviour.

[41] Provine RR (1986). Yawning as a stereotyped action pattern and releasing stimulus. Ethology.

[42] Kapitány R, Nielsen M (2017). Are yawns really contagious? A critique and quantification of yawn contagion. Adapt. Hum. Behav. Physiol..

[43] Bates D, Mächler M, Bolker B, Walker S (2015). Fitting linear mixed-effects models using lme4. J. Stat. Softw..

[44] Dobson AJ (2002). An Introduction to Generalized Linear Models.

[45] Barr DJ, Levy R, Scheepers C, Tily HJ (2013). Random effects structure for confirmatory hypothesis testing: keep it maximal. J. Mem. Lang..

[46] Ben-Shachar MS, Lüdecke D, Makowski D (2020). effectsize: Estimation of effect size indices and standardized parameters. J. Open. Source. Softw..

[47] Massen JJ, Church AM, Gallup AC (2015). Auditory contagious yawning in humans: an investigation into affiliation and status effects. Front. Psychol..

[48] Bergman TJ, Beehner JC (2015). Measuring social complexity. Anim. Behav..

[49] Deputte BL (1994). Ethological study of yawning in primates: Quantitative analysis and study of causation in two species of Old World monkeys (*Cercocebus albigena* and *Macaca fascicularis*). Ethology.

[50] Duranton C, Gaunet F (2016). Behavioural synchronization from an ethological perspective: Overview of its adaptive value. Adapt. Behav..

[51] Clay Z, De Waal FB (2013). Bonobos respond to distress in others: consolation across the age spectrum. PLoS ONE.

[52] Lakin JL, Jefferis VE, Cheng CM, Chartrand TL (2003). The chameleon effect as social glue: Evidence for the evolutionary significance of nonconscious mimicry. J. Nonverbal Behav..

[53] Palagi E, Celeghin A, Tamietto M, Winkielman P, Norscia I (2020). The neuroethology of spontaneous mimicry and emotional contagion in human and non-human animals. Neurosci. Biobehav. Rev..

[54] Preston SD, De Waal FB (2000). Empathy: Its ultimate and proximate bases. Behav. Brain Sci..

