# Supplementary material for: First evidence of yawn contagion in a wild monkey species
Source: Sci Rep. 2021 Sep 9;11:17957. doi: 10.1038/s41598-021-96423-3 (PMC8429631; doi:10.1038/s41598-021-96423-3)
Supplement: Supplementary file 1 — Supplementary Information 1. [file 41598_2021_96423_MOESM1_ESM.pdf]

SUPPLEMENTARY MATERIAL

YAWN CONTAGION GOES WILD: FIRST EVIDENCE IN A FREE-RANGING MONKEY SPECIES

Alessandro Gallo[a], Anna Zanoli[a], Marta Caselli[a], Elisabetta Palagi[b,c*]/Ivan Norscia[a,b *]

[a] Department of Life Sciences and Systems Biology, University of Torino, Via Accademia Albertina 13, 10123 Torino (Italy)

[b] Natural History Museum, University of Pisa, Via Roma 79, 56011 Calci (Pisa, Italy)

[c] Unit of Ethology, Department of Biology, University of Pisa, Via Alessandro Volta 6, 56126 Pisa (Italy)

Video S1 - Legend

Yawn contagion between gelada males from two different OMUs on the Kundi pleateau Ethiopia

Raw data: attached csv file

Supplementary Table S1 - Results of the GLMMs performed considering 1min ($GLMM_1$) and 2min ($GLMM_2$) time window from the triggering yawn. Fixed factors: Perception (Yes/No), Group membership (Same/Different), Distance (Proximity/Non-proximity), Time slot (09:01-12:00/12:01-15:00), Sex Trigger (Male/Female), Sex Responder (Male/Female), Yawn duration, Yawn type (Covered Teeth/Uncovered Teeth/Uncovered Gums), Yawn vocalization (Presence/Absence). Random factors: group identity of the dyad. $GLMM_1$: The full model is not significantly different from the null model, therefore no yawn contagion could be detected within 1min from the triggering stimulus. $GLMM_2$: the full model is significantly different from the null model. Perception, Group membership and responder sex had a significant effect as it occurs in the 3-min time window (Table 1 in the main text of the article).

Table S1

GLMM$_1$ performed on 1min time window.

Dependent variable=yawning performed by a subject within one minute from others' yawns (binomial: present=1; absent=0). Full-model not significantly different from null-model: $\chi 2=16.776$, df = 10, p=0.080.

GLMM$_2$ performed on 2min time window.

Dependent variable=yawning performed by a subject within two minutes from others' yawns (binomial: present=1; absent=0). Full- versus null-model: $\chi 2=29.895$, df = 10, p<0.001.

**Fixed Effects**	**Estimate**	**SE**	***z value***	***p value***
Intercept	-0.894	2.185	-0.409	a
Perception (Yes)[b,c]	2.505	0.974	2.572	**0.010**
Group membership (Same)[b,c]	-2.937	1.304	-2.253	**0.024**
Distance (Non-proximity)[b,c]	-1.044	1.190	-0.877	0.381
Time slot (12:01-15:00)[b,c]	1.233	0.935	1.319	0.187
Sex Trigger (Female)[b,c]	0.210	0.916	0.230	0.818
Sex Responder (Female)[b,c]	-1.384	0.648	-2.136	**0.033**
Yawn duration	0.418	0.847	0.494	0.622
Yawn type (Uncovered gums)[b,c]	-0.292	0.790	-0.370	0.711
Yawn type (Uncovered teeth)[b,c]	-1.639	1.230	-1.333	0.182
Yawn vocalization (Presence)[b,c]	-1.550	1.082	-1.433	0.152

[a]*Not shown as not having a meaningful interpretation.*

[b]*Estimate ± SE refers to the difference of the response between the reported level of this categorical predictor and the reference category of the same predictor.*

[c] *These predictors were dummy coded, with the "Perception (NO)", "Group membership (Different)", "Distance (Proximity)", "Time slot (09:01-12:00)", "Sex Trigger (Male)", Sex Responder (Male)", "Yawn vocalization (Absent)", "Yawn type (Covered teeth)", being the reference categories.*
